# A metagenomic study of diet-dependent interaction between gut microbiota and host in infants reveals differences in immune response

**DOI:** 10.1186/gb-2012-13-4-r32

**Published:** 2012-04-30

**Authors:** Scott Schwartz, Iddo Friedberg, Ivan V Ivanov, Laurie A Davidson, Jennifer S Goldsby, David B Dahl, Damir Herman, Mei Wang, Sharon M Donovan, Robert S Chapkin

**Affiliations:** 1 Training Program in Biostatistics, Bioinformatics, Nutrition and Cancer, Texas A&M University, 155 Ireland Street, College Station, TX 77843, USA; 2 Department of Statistics, Texas A&M University, 155 Ireland Street, College Station, TX 77843, USA; 3 Department of Microbiology, Miami University, 700 East High St, Oxford, OH 45056, USA; 4 Program in Integrative Nutrition and Complex Diseases, Texas A&M University, College Station, TX 77843, USA; 5 Veterinary Physiology and Pharmacology, Texas A&M University, College Station, TX 77843, USA; 6 Division of Hematology and Oncology, Winthrop P Rockefeller Cancer Institute, University of Arkansas for Medical Sciences, 4301 W. Markham St, Little Rock, AR 72205, USA; 7 Department of Food Science and Human Nutrition, 905 S. Goodwin Avenue, University of Illinois, Urbana, IL 61801, USA; 8 Department of Microbial and Molecular Pathogenesis, Texas A&M Health Science Center, 8441 State Hwy 47, Bryan, TX 77807, USA; 9 Computer Science and Software Engineering, Miami University, 700 East High St, Oxford, OH 45056, USA

## Abstract

**Background:**

Gut microbiota and the host exist in a mutualistic relationship, with the functional composition of the microbiota strongly affecting the health and well-being of the host. Thus, it is important to develop a synthetic approach to study the host transcriptome and the microbiome simultaneously. Early microbial colonization in infants is critically important for directing neonatal intestinal and immune development, and is especially attractive for studying the development of human-commensal interactions. Here we report the results from a simultaneous study of the gut microbiome and host epithelial transcriptome of three-month-old exclusively breast- and formula-fed infants.

**Results:**

Variation in both host mRNA expression and the microbiome phylogenetic and functional profiles was observed between breast- and formula-fed infants. To examine the interdependent relationship between host epithelial cell gene expression and bacterial metagenomic-based profiles, the host transcriptome and functionally profiled microbiome data were subjected to novel multivariate statistical analyses. Gut microbiota metagenome virulence characteristics concurrently varied with immunity-related gene expression in epithelial cells between the formula-fed and the breast-fed infants.

**Conclusions:**

Our data provide insight into the integrated responses of the host transcriptome and microbiome to dietary substrates in the early neonatal period. We demonstrate that differences in diet can affect, via gut colonization, host expression of genes associated with the innate immune system. Furthermore, the methodology presented in this study can be adapted to assess other host-commensal and host-pathogen interactions using genomic and transcriptomic data, providing a synthetic genomics-based picture of host-commensal relationships.

## Background

The gut microbiota has profound effects on the health and wellness of the host. For example, studies in germ-free piglets clearly illustrate altered intestinal growth [1], digestive enzyme activity [2] and development of the gut-associated lymphoid tissue [3]. Molecular-level studies, enabled by metagenomic, metatranscriptomic and metaproteomic analytical techniques, are reshaping our understanding of how the gut microbiome modulates gastrointestinal morphological, immune development [1-4], gene expression [5], and the biology of the host in general [6,7]. Although many studies have shown an effect of diet on the infant microbiota [8-10], little is known of the genome and transcriptome-level cross-talk between the developing infant gut and the colonizing microbiota. At birth, the intestinal tract of the human infant is functionally immature and sterile. Accordingly, the early neonatal period is a critical phase for both intestinal digestive development as well as colonization by the commensal microbiota.

The human intestine is lined by epithelial cells that process nutrients and provide the first line of defense against food antigens and pathogens. Approximately one-sixth of intestinal epithelial cells are shed (exfoliated) daily [11]. This corresponds to the daily exfoliation of 10^8 ^to 10^10 ^cells [11]. Because colonization of the intestine with non-pathogenic (commensal) microbiota is vital for neonatal intestinal development [1,2,5], it is important to understand how epithelial cells and the microbial ecosystem are modulated by diet. Therefore, our ongoing efforts have been directed at understanding the regulation of neonatal development by components present in human milk. Our initial work isolated exfoliated eukaryotic 'host' cell mRNA from feces, which contain sloughed (intact) intestinal cells, to determine which gene combinations best distinguish the feeding groups. We previously reported that two- and three-gene combinations provide classifiers with potential to non-invasively identify discriminative molecular signatures in the developing human neonate [12]. Specifically, linear discriminant analysis (LDA) was used to identify the best single, two and three-gene combinations for classifying the experimental treatments. LDA is a technique developed for the purpose of statistical pattern recognition [13]. Using a selected list of features, it aims at constructing a discriminating hyperplane that separates the observations from two different classes with a minimum misclassification error. Therefore, gene sets or combinations are identified in response to treatments, as opposed to simply determining up- or down-regulated mRNA expression levels. It is important to emphasize that, previously, our main objective was to identify candidate biomarker genes [12], and not to probe for interrelationships between the host gut transcriptome and metagenome. In particular, we focused on two major issues: finding groups of genes that discriminate between breast-fed and formula-fed babies, in terms of LDA classification; and identifying potential 'master' regulators as defined by the statistical properties of the non-linear coefficient of determination (CoD). The current manuscript uses a linear model, canonical correlation analysis (CCA), in order to detect interdependencies between the host intestinal transcriptome and the metagenome in healthy full-term infants.

We now present a systematic and statistically rigorous analytical framework for the simultaneous examination of both host and microbial responses to dietary/environmental components in the early neonatal period. Specifically, we tested the hypothesis that the integration of infant (host) epithelial cell transcriptome and functionally profiled microbiome can be used to suggest important regulatory pathways of the microbiome affecting intestinal development in the first few months of life. Initially, we examined the multivariate correlation structures between host intestinal mRNA gene expression levels and functional annotations in genes in the gut metagenome of exclusively breast-fed (BF) and formula-fed (FF) infants at three months of age. Interestingly, we found that the microbiome of BF infants is significantly enriched in genes associated with virulence functionality. Furthermore, we demonstrate a multivariate correlation between the gut flora genes associated with bacterial pathogenicity and the expression of host genes associated with immune and defense mechanisms. In addition, the operational taxonomic unit (OTU) composition and genetic potential of the microbiota differed between BF and FF infants. Our findings suggest that human milk promotes the mutualistic crosstalk between the mucosal immune system and the microbiome in the maintenance of intestinal homeostasis.

## Results

A total of six mothers of BF infants and six mothers of FF infants were recruited for the study. Briefly, stool samples from each infant were collected, and microbial DNA was extracted and sequenced. Additionally, mRNA was isolated from stool containing host gut exfoliated epithelial cells and processed for microarray analysis [12]. These two concurrent operations provided the raw microbial metagenomic and host transcriptomic data. We subsequently analyzed the sequence and microarray data independently and then simultaneously to identify multivariate correlations between the gut epithelium transcriptome and the microbial metagenome. The procedure is outlined in Additional file 1 (see Materials and methods for details). As shown in Table 1, infant and mother data were appropriately balanced across FF and BF infants.

**Table 1:** Infant growth characteristics

	Breast-fed (BF)	Formula-fed (FF)
Sample size	6	6
Maternal age	30.0 ± 4.6	30.7 ± 5.9
Parity	2.0 ± 0.0	2.1 ± 0.6
Infant gender	5 male/1 female	4 male/2 female
Length at birth (cm)	53.2 ± 3.1	51.0 ± 2.5
Body weight (kg)		
At birth	3.79 ± 0.50^a^	3.46 ± 0.20^a^
At month 1	4.98 ± 0.73^b^	4.61 ± 0.65^b^
At month 2	6.43 ± 0.73^c^	5.66 ± 0.85^c^
At month 3	7.02 ± 0.72^d^	6.45 ± 0.96^d^
BF or FF diet intake (ml/kg/day)		
At month 1	166.0 ± 18.3^a^	162.5 ± 28.4^a^
At month 2	127.6 ± 19.5^a^	138.5 ± 14.3^a,b^
At month 3	129.0 ± 20.1^b^	134.8 ± 9.5^b^

Values are means ± standard deviation. Superscripts indicate significant differences over time, *P *= 0.001.

### Effect of diet on host transcriptional responses

**Figure 1:**
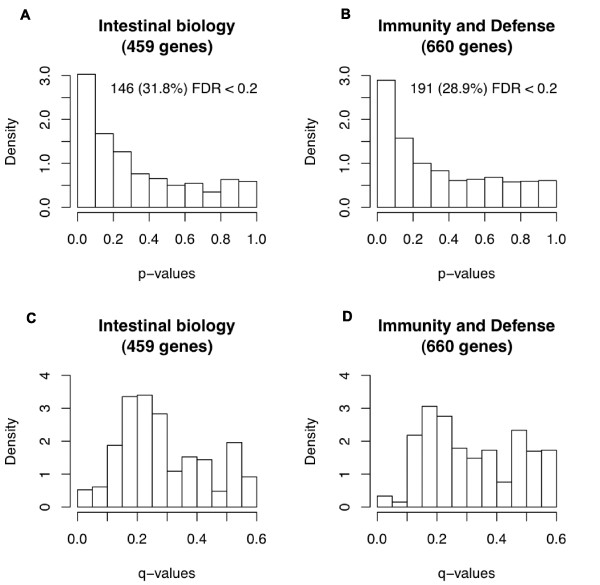
**Effect of diet on host transcriptional responses**. Genes known *a priori *to be involved in intestinal biology or immunity and defense mechanisms were enriched for differential expression between BF and FF infants. **(a-d) **The distribution of *P*-values (a,b) and the distribution of q-values (c,d). (a,c) Intestinal biology: 459 genes known to be related to intestinal biology passed the quality control measures and were tested for differential expression between the BF and FF infants - 146/459 genes (32%) had FDR corrected q-values <0.2. (b,d) Immunity and defense: 660 genes known to be related to immunity and defense that passed the quality control measures and tested for differential expression between the BF and FF infants - 191/660 genes (29%) had FDR corrected q-values <0.2.

As shown in Additional file 2, in general, FF host cell samples exhibited lower gene expression values relative to BF host cell samples. These data are consistent with a Rhesus monkey study, in which formula-feeding down-regulated overall intestinal gene expression relative to breast-fed monkeys [14]. Genes from two data subsets - 459 intestinal biology-related genes and 660 immunity and defense-related genes - were tested for differential expression between BF and FF infants using a permutation test with a false discovery rate (FDR) [15] multiple testing correction. As seen in Figure 1, the genes expected *a priori *to be responsive to diet were enriched for differential expression. This suggests our *a priori *knowledge allowed for the detection of relevant genes. As a follow-up examination, 146 of 459 intestinal biology genes and 191 of 660 immunity and defense genes exhibiting an FDR q-value <0.2 were subjected to an independent Gene Ontology (GO) [16] analysis. Since these sets were chosen *a priori *for related biological functionality, GO enrichment analysis was performed with respect to the original gene sets (459 and 660 genes). Additional file 3 lists the GO categories for genes in the list and the GO analysis p-values and q-values, indicating that the categories are significantly affected by treatment. As expected, we did not detect enrichment of GO categories on which the sets are based. For example, genes with GO immune response attributes were not enriched with respect to the immunity and defense gene set since these are exactly the types of genes comprising this set. Nonetheless, Additional file 3 provides a general characterization of the genes. Interestingly, there was no enrichment of differential expression in genes related to the cell death biological process.

### Effect of diet on the gut microbiome

#### Taxonomical analysis

**Figure 2:**
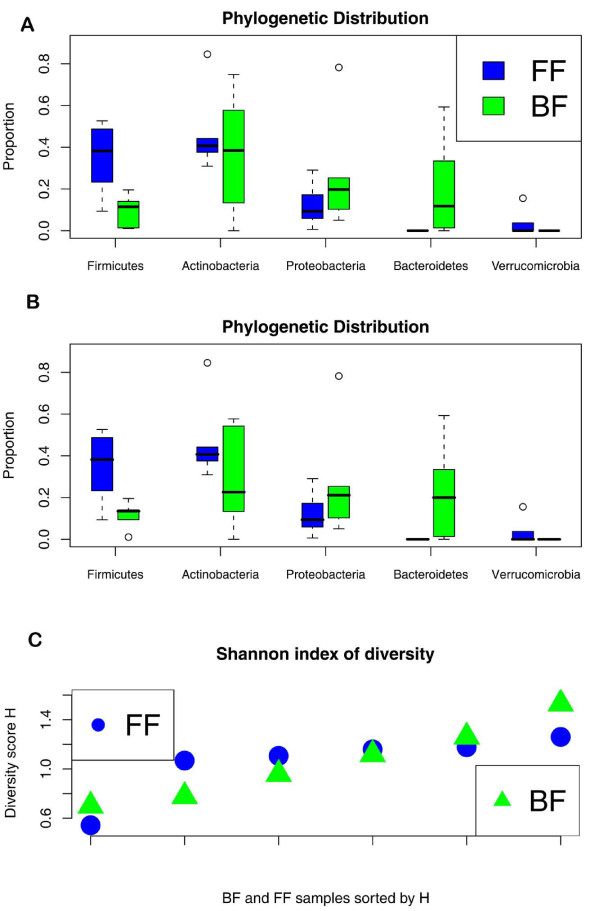
**Effect of diet on infant microbiota**. BF (breast-fed) infants (green) exhibited more heterogeneity than FF (formula-fed) infants (blue) with respect to phylogenetic composition. **(a) **Taxon assignment (phylum level) variability for BF and FF samples using 16S rRNA alignments to GreenGenes (see Materials and methods). A diet label permutation test using the statistic ∑_s _|∑_iεBF _p_is_/6 - ∑_iεFF _p_is_/6|, where s indexes phylum and iεBF and iεFF denote that sample i is BF or FF infant, respectively, and p denotes the associated taxon proportion, rejected the null hypothesis that variability in phylogenetic composition was unrelated to BF/FF status with a *P*-value of 0.011. **(b) **Taxon assignments for all the shotgun reads (not just 16S rRNA homologs) using PhymmBL [17]. **(c) **Shannon-Weiner index for BF and FF infants, indicating alpha-diversity for each sample.

As seen in Figure 2a,b, there were substantial differences in the taxonomic distribution of identifiable 16S rRNA in FF and BF infant microbiota. The FF infant microbiota was very homogeneous in phylum-level distributions. Specifically, there were approximately an equal proportion of Firmicutes and Actinobacteria (about 40% each), with the remaining bacteria composed of predominantly Proteobacteria. One FF infant microbiota was a clear outlier and was dominated by Actinobacteria. In comparison, BF infants were much more heterogeneous with respect to their phyla composition. The microbiota of three BF infants were dominated by Actinobacteria, one was dominated by Proteobacteria, one was dominated by Bacteroidetes, and one was very balanced across the phyla. As seen in Figure 2c, with the exception of the outlying FF infant microbiota, the BF infant microbiota exhibited a higher alpha-diversity than the FF infant microbiota as quantified by the Shannon-Wiener index.

To confirm our findings, we used PhymmBL to taxonomically classify shotgun sequence reads. PhymmBL [17] is a classification approach for metagenomics data that uses interpolated Markov models (IMMs) and Basic Local Alignment Search Tool (BLAST) to taxonomically classify DNA sequences. The reads were assigned to phyla as summarized in Additional files 4 and 5. While there was general agreement between the 16S-based analysis and the whole shotgun-reads-based analysis, we did identify some inconsistencies. These corresponded to similar discrepancies found in Koenig *et al*. [18], and are possibly due to under-representation of 16S rRNA from Actinobacteria. Overall, both analyses are consistent with a previous report indicating a high level of Actinobacteria and Proteobacteria in infants at 3 months of age [18].

#### Functional analysis

**Figure 3:**
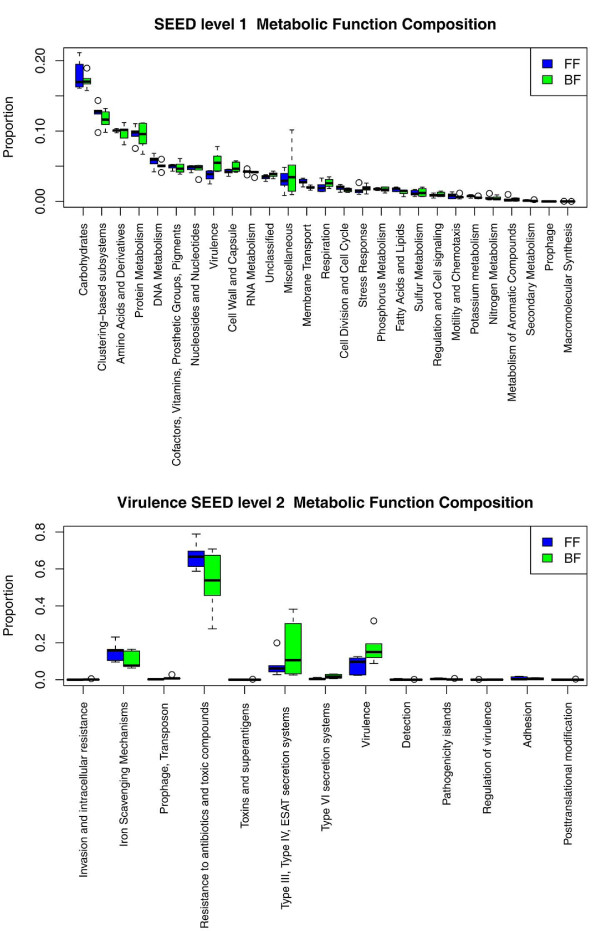
**Functional analysis of metagenomic data**. Top panel: SEED level 1 categories for which all BF or all FF samples had at least 200 reads mapped. At least 2% of the total number of mapped reads were tested for differences between BF (breast-fed) infants (green) and FF (formula-fed) infants (blue). A permutation test on the test statistic ∑_iεBF _p_i_/6 - ∑_iεFF _p_i_/6, where iεBF and iεFF denote that sample i is BF or FF infant, respectively, and p denotes the associated taxon proportion, was performed. The FDR corrected q-value for the virulence category was 0.058. Bottom panel: differences between BF and FF infants in the SEED level 2 virulence assignment (within the SEED level 1 virulence category) was assessed using a permutation test on the test statistic ∑_s _|∑_iεBF _p_is_/6 - ∑_iεFF _p_is_/6|, where s indexes the SEED level 2 virulence categories, and *P *= 0.0140.

To investigate the diet-driven variation in the gut metagenome, the shotgun sequenced data were aligned using Rapid Annotation using Subsystems Technology (MG-RASTv2) against the SEED subsystems database [19]. Genes in SEED were annotated using a three-level biological-function ontology, with level 1 being the most general, and level 3 being the most specific. The gene-level annotation describes the type of subsystem to which each gene belongs. A subsystem 'represents the collection of functional roles that make up a metabolic pathway, a complex (e.g., the ribosome), or a class of protein' [20]. Figure 3 (upper panel) shows the frequency of SEED functional terms in the BF and FF microbiomes. A permutation test was used to examine if the relative abundance of the functional category varied between BF and FF infants for SEED level 1 categories (with at least 200 reads comprising a minimum of 2% of all reads for all BF or FF samples). Upon correction for multiple testing using the FDR [15], the virulence characteristics of the microbiota were the only potentially responsive characteristics with respect to diet composition (q-value = 0.058, all other q-values >0.3). Strengthening this finding, a permutation test has shown that the relative proportion of SEED level 2 characteristics as a whole within the SEED level 1 virulence category (Figure 3) differed between BF and FF infants (*P*-value = 0.014). Four SEED level 2 virulence categories comprised the overwhelming majority of sequence reads, with an average number of sequence reads of 245 for each category for each infant. The four virulence characteristics included 'iron scavenging mechanisms', 'resistance to antibiotics and toxic compounds', 'Type III, Type IV, early secreted antigenic target (ESAT) secretion systems', and 'virulence'. The first three were noted as being associated with invasiveness.

The virulence characteristics of the microbiota were the only functional characteristics that appeared to differ between the BF and FF infants. However, we tested the remaining 36 of 149 non-virulence SEED level 2 categories in which all the FF or BF samples had at least 100 reads comprising a minimum of 0.5% of the total number of reads. All *P*-values were greater than 0.05, and we did not, therefore, calculate q-values or examine the non-virulence SEED level 2 classifications. For the 84 of 584 SEED level 3 categories (consisting of sub-classifications of 'accessory colonization factor', 'Ton and Tol transport systems', 'type 1 pili (mannose-sensitive fimbriae)', 'the *Streptococcus pyogenes *Virulom', 'bacterial cyanide production' and 'tolerance mechanisms') for which all the FF or BF samples had at least 50 reads comprising at least 0.1% of the total number of reads, a permutation test was used to examine if the relative abundance of the functional category varied between BF and FF infants. Some *P*-values were <0.05, but no q-values were <0.10 upon FDR correction and so SEED level 3 classifications were not examined.

### Interactions between the gut microbiome and the host transcriptome

For the purpose of uncovering potential symbiotic gut microbial-host metabolic interactions, a variation of CCA was used to examine the multivariate structure between the most promising virulence characteristics of the microbiota (resistance to antibiotics and toxic compounds, Type III, Type IV, ESAT secretion systems, and iron scavenging mechanisms) and host transcriptome data sets. For each gene triple selected from a transcriptome set and analyzed with the metagenomic virulence variables (as described in Materials and methods) there were three canonical correlations. Canonical correlations represent the strongest (ordered) correlations created between linear composites (called canonical variates) of the gene triples with the metagenomic variables (subject to some optimization constraints involving the independence and variation of canonical variates). Hence, they represent the strength of the linear multivariate relationship between the particular host gene triple being analyzed and the microbiome virulence variables [21-23].

**Figure 4:**
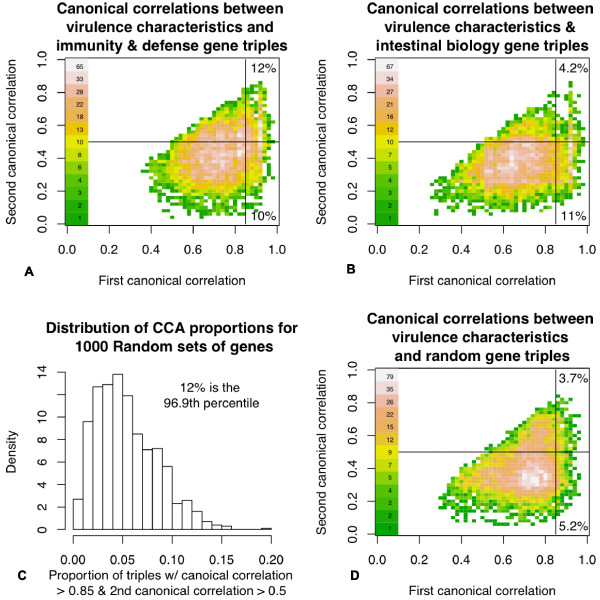
**First and second canonical correlations between host gene sets and microbial virulence characteristics**. Horizontal lines in the density plots are at 0.5, and the vertical lines are at 0.85. These cutoffs were chosen arbitrarily to emphasize enrichment in the upper-right quadrant of the plot that is suggestive of increased multivariate structure as identified by CCA. **(a) **First and second canonical correlations between triples of immunity and defense genes and virulence variables are shown. There are increased canonical correlations in the upper-right corner of the plot, suggesting an enriched multivariate relationship between the immunity and defense genes and microbiome virulence characteristics as compared to, for example, the set of random genes shown in (d). **(b) **Intestinal biology genes did not show the same level of enrichment of canonical correlations as the immunity and defense genes. **(c) **We analyzed 1,000 random sets each containing 660 genes in an analogous manner to the immunity and defense gene analysis (a). Of these, 969 random sets resulted in less than 12% of analyzed gene triples having first canonical correlation >0.85 and second canonical correlation >0.5. **(d) **An example random gene CCA plot. Additional examples are given in Additional file 6.

Figure 4 shows the distribution of first and second canonical correlations for triples of 100 of 459 intestinal biology genes with the smallest *P*-values for differential expression between BF and FF and triples of the 100 of 660 immunity and defense genes with the smallest *P*-values for differential expression between BF and FF. In addition, the same distribution is shown for an example set of 100 of 660 random genes that have the smallest *P*-values for differential expression between BF and FF infants (additional example and representative plots of random gene sets are described in Additional file 6). Finally, based on 1,000 random gene sets analyzed in an analogous manner to the example random gene set, the distribution of the proportion of the random gene set triples that have a canonical correlation >0.85 and a second canonical correlation >0.5 is shown (Figure 4). The 100 genes with the best *P*-values in the random gene set were used so the number of triples for each gene assigned was the same across data sets, and the results could be compared to the *a priori *knowledge gene sets. The analyses indicate that the large majority of gene triples scored comparably weakly in terms of canonical correlations with virulence characteristics. However, the SEED-categorized immunity and defense gene triples (Figure 4a), and to a lesser extent the intestinal biology gene triples (Figure 4b), exhibited an enrichment of gene triples indicating a correlation and probable empirical relationship with the microbiota virulence characteristics. The enrichment of immunity and defense gene multivariate relationships relative to the random gene sets is shown in Figure 4c,d. Specifically, 12% of immunity and defense gene triples with a first canonical correlation >0.85 and second canonical correlation >0.5 were associated with the 96.9th percentile of the 1,000 random gene set comparable percentages. This enrichment suggests that there are indeed relationships between immunity and defense genes of the host and the virulence characteristics of the microbiome, as might be expected since these are the genes considered most likely to respond to microbiota virulence characteristics.

**Figure 5:**
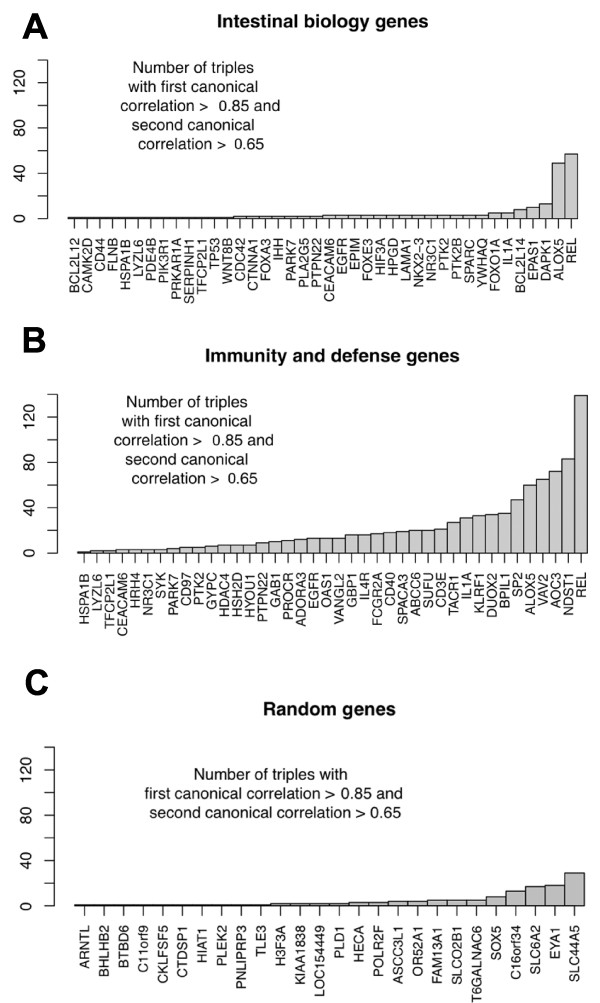
**Frequency of host genes appearing in triples**. Sets of gene triples were included when the first canonical correlation was at least 0.85 and the second canonical correlation was at least 0.65. These levels were chosen arbitrarily to represent strong multivariate structure as identified by CCA. Genes were ranked by their prevalence of top performing triples. This provided a qualitative profile to select genes that empirically show the strongest potential for being related to the virulence characteristics of the microbiome. **(a,b) **Genes related to immunity and defense far outperformed the other functional categories. For example, the best two performing intestinal biology genes were in fact also co-listed as immunity and defense genes. **(c) **In contrast, randomly selected genes did not display any strong multivariate structure with respect to the virulence characteristics of the microbiome.

On the basis of the canonical correlations from the gene triple CCA analyses, individual expressed host genes were ranked relative to their empirical multivariate relationship with the frequency of genes in the metagenome. To construct a list of the most promising host genes, we examined the proportion of gene triples whose first canonical correlation coefficient was at least 0.85 and whose second canonical correlation was at least 0.5. These were chosen to highlight the enrichment of first and second canonical correlation scores observed in the northeast quadrant for the immunity and defense gene set (Figure 4a). The resulting counts for the immunity and defense genes, the intestinal biology genes, and the example random gene set are shown in Figure 5 (additional example and representative plots of random gene sets are available in Additional file 7). The genes showing the strongest empirical multivariate relationship with the metagenomic-derived virulence variables were from the immunity and defense gene set.

**Figure 6:**
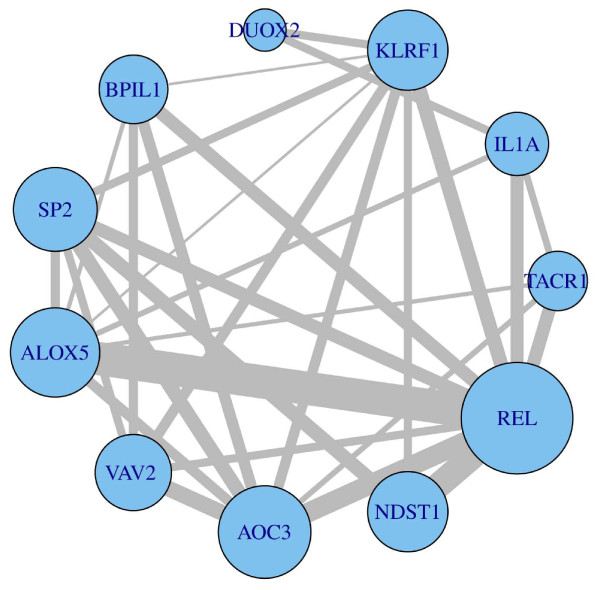
**Relative performance of the top 11 immunity and defense host genes and virulence characteristics**. Data were assessed by the ranking described in Figure 5 with respect to multivariate association between annotated genes and the virulence characteristics of the microbiome. The size of the nodes reflects the number of triples of genes whose first canonical correlation was at least 0.85 and whose second canonical correlation was at least 0.5. The thickness of the edges connecting the nodes reflects the number of triples whose first canonical correlation was at least 0.85 and whose second canonical correlation was at least 0.5. This plot summarizes the potential relationships between genes with respect to the virulence characteristics of the microbiome. *ALOX5*, arachidonate 5-lipoxygenase; *AOC3*, amine oxidase, copper containing 3 (vascular adhesion protein); *BPIL1*, bactericidal/permeability-increasing protein-like 1; *DUOX2*, dual oxidase 2; *IL1A*, interleukin 1 alpha; *KLRF1*, killer cell lectin-like receptor subfamily F, member 1; *NDST1*, N-deacetylase/N-sulfotransferase (heparan glucosaminyl) 1; *REL*, v-rel reticuloendotheliosis viral oncogene homolog; *SP2*, Sp2 transcription factor; *TACR1*, tachykinin receptor 1; *VAV2*, vav 2 guanine nucleotide exchange factor.

The 11 most promising identified host genes are listed along with their functional annotation and related biological response; *VAV2 *(angiogenesis), *ALOX5 *(inflammatory response), *SP2 *(transcription factor), *BPIL1 *(bacteriocidal), *DUOX2 *(peroxidase generation), *KLRF1 *(cytotoxicity), *IL1A *(inflammatory response); *AOC3 *(vascular adhesion), *NDST1 *(inflammation and mucosal defense), *REL *(intestine proliferation and apoptotic homeostasis) and *TACR1 *(gut motility). As can be seen, most of these genes are associated with immune response. The relative gene expression levels in BF versus FF infants following a 3-month feeding period are shown in Table 2. Since canonical correlations are assigned to triples rather than single genes, we also examined which genes together exhibited the most promising multivariate relationship to the microbiome variables. Figure 6 shows which genes most frequently had the best canonical correlations (size of node) and which gene pairs together in the same triple most frequently had the best canonical correlations (size of edge). This visualization provides a view of the synergistic strength between genes with respect to improving the multivariate microbiome relationship characteristics.

**Table 2:** Relative gene expression levels in breast-fed (BF) versus formula-fed (FF) infants following a 3-month feeding period

Gene	BF/FF	*P*-value	q-value
*TACR1*	1.80	0.0189	0.1670
*REL*	1.62	0.0047	0.1026
*DUOX2*	1.45	0.0215	0.1670
*VAV2*	1.36	0.0088	0.1404
*NDST1*	0.79	0.0103	0.1477
*AOC3*	0.78	0.0202	0.1670
*SP2*	0.76	0.0030	0.0860
*IL1A*	0.71	0.0089	0.1389
*ALOX5*	0.69	1.40E-05	0.0008
*BPIL1*	0.37	1.43E-05	0.0008
*KLRF1*	0.35	3.16E-05	0.0015

Fold change represents relative expression level in BF divided by FF infants for the 11 genes exhibiting the strongest multivariate relationships to microbiota virulence characteristics.

## Discussion

Our ongoing efforts are directed at understanding the regulation of neonatal gastrointestinal development by components present in human milk. The neonatal gastrointestinal tract undergoes pronounced structural and functional changes [24], which are influenced by diet [25,26]. For example, there is a stronger trophic response to human milk than formula, suggesting that the bioactive components in human milk are important for gastrointestinal development [27,28]. Furthermore, the composition of the neonatal microbiota undergoes successional changes, which is profoundly influenced by diet [8-10]. Given the need to better understand neonatal gastrointestinal health and development, we recently demonstrated that host gene set combinations provide discriminative molecular signatures for distinguishing BF versus FF infants [12]. However, no studies to date have attempted to systematically integrate genomic data from both the infant (host mucosa) and gut microbiome. Therefore, the goal of this study was to assess host gene-diet interactions within the context of the structure and operations of gut microbial communities. As part of this effort, we examined multivariate correlation structures between host intestinal mRNA gene signatures and biological processes/metabolic pathways in the gut metagenome of exclusively BF and FF infants at 3 months of age. Microbial composition of the same stool samples was assessed by metagenomic pyrosequencing, thereby providing a picture of the integrated gut/microbial ecosystem. Consistent with previous reports [8-10], the bacterial microbiome phylogenetic profiles strongly characterized the two groups of infants (FF and BF).

There are at least two viable approaches for uncovering the interdependencies between the intestinal transcriptome and the microbiome of the developing infant gut with respect to diet (BF versus FF). The first approach is to evaluate each data set independently on a variable-by-variable basis, and then produce one feature list for each data set in order to explore possible qualitative relationships between the feature lists. For example, Mulder and colleagues [29] performed traditional metagenomic and differential expression analyses and highlighted plausible relationships between the prominent results from each of the two analyses. The second approach is to analyze the two data sets simultaneously under an analytical framework designed to assess the 'many to many' multivariate relationship between the two variable sets. This provides a quantifiable and objective way to discover and evaluate multivariate relationships between data sets. For example, CCA has been used to evaluate the multivariate relationships between salt-water microbiomes and environmental variables, such as salinity, sample depth, water column depth, temperature and chlorophyll content [30]. We used elements of both approaches to examine potential relationships underlying interactions between the microbiota and the host transcriptome in the developing infant gut. First, an independent analysis of each data set was used to reduce the number of variables under consideration to a computationally tractable number that could be readily assessed by the methods we present. Secondly, based on the previous findings, an analytical multivariate assessment of the microbiome/transcriptome structure was used to inform our qualitative interpretation of the connections between the two.

By initially examining the metagenomic data, we noted that the 'resistance to antibiotics and toxic compounds', 'Type III, Type IV, ESAT secretion systems', 'iron scavenging mechanisms' and 'virulence' characteristics of the microbiome exhibited evidence of differential sensitivity to breast milk as compared to formula. Specifically, while other microbiome characteristics did not provide strong evidence of differentiation between BF and FF infants upon adjustment for multiple testing, virulence-related microbial genes remained strong. Therefore, we focused our transcriptomic analyses on host immunity and defense-associated genes. Additionally, since we were studying the developing human gut, genes known to be involved in intestinal biology were also examined. Our initial differential expression analysis suggested that our prior knowledge gene sets were targeting relevant gene sets.

Next, the metagenomic and transcriptomic data sets were analytically combined in a multivariate analysis that allowed us to assess the strength of the multivariate relationship between the virulence variables of the microbiome and the gut transcriptome genes under examination. Ranking of the best performing genes under consideration indicated that the strongest multivariate relationship with the virulence characteristics were immunity and defense genes. The credibility of this finding was supported by the *a priori *expectation that the strongest relationships with the virulence characteristics of the microbiome would be the immunity and defense genes, and the comparison to 1,000 random gene sets to which the immunity and defense gene set ranked in the 96.9th percentile with respect to the measure of transcriptome/microbiome multivariate strength. While the intestinal biology showed some strong multivariate relationships to the microbiome virulence characteristics, they were not unusual compared to the random gene sets, and certainly not as strong as those of the immunity and defense genes (Figure 4).

By adapting CCA outcomes, we identified a subset of 11 immunity and mucosal defense-related genes (*REL*, *NDST1*, *AOC3*, *VAV2*, *ALOX5*, *SP2*, *BPIL1*, *DUOX2*, *KLRF1*, *IL1A*, and *TACR1*) that exhibited evidence of a multivariate relationship with microbiome virulence characteristics (Figure 6). Although it is premature to assign cause and effect, we conjecture these genes are reacting concordantly in response to microbial conditions. It is interesting to note that genes that modulate gut motility (tachykinin receptor 1, *TACR1*) [31] and bacterial-mediated reactive oxygen species signaling/epithelial homeostasis (dual oxidase 2, *DUOX2*; Vav 2 guanine nucleotide exchange factor, *VAV2*; v-rel reticuloendotheliosis viral oncogene homologue, *REL*) [32-36], were up-regulated in BF versus FF infants (Table 2). In contrast, genes that prime mucosal inflammatory responses (killer cell lectin-like receptor subfamily F-member 1, *KLRF1*; bactericidal/permeability-increasing protein-like 1, *BPIL1*; arachidonate 5-lipoxygenase, *ALOX5*; interleukin 1 alpha, *IL1A*; vascular adhesion protein 1, *AOC3*) [37-39], were down-regulated in BF versus FF infants. Collectively, these data are consistent with previous findings that breastfeeding facilitates the adaptive, functional changes required for optimal transition from intrauterine to extrauterine life [27]. Our systems-level analyses support previous studies showing that human milk optimally promotes the mutualistic crosstalk been the mucosal immune system and the microbiome in the maintenance of intestinal homeostasis [8,9,25,27,28].

## Conclusion

We have identified a subset of 11 immunity/defense-related genes that exhibited evidence of a multivariate relationship with microbiome virulence and invasiveness characteristics. To our knowledge, this is the first time an assessment of the multivariate relationship between the microbiome and the host transcriptome has been used to identify intestinal genes potentially important in microbiome regulatory pathways and the integrative gut development process. Arguably, by examining the multivariate structure underlying the microbiome and gut transcriptome, our approach leverages richer and fuller information content compared to analyses focusing on single data sets (for example, only host transcriptome data, or only microbiome data) and only single variables (for example, gene by gene differential expression testing). Our study provides a systematic and statistically rigorous analytical framework for the examination of both host and microbial responses to dietary/environmental components in the early neonatal period. Finally, the novel methodology described here for multivariate correlation analysis of host transcriptome and microbiome can be successfully applied to a large variety of host/microbial commensal studies. The use of CCA can support the formulation of hypothesis-based studies by accurately identifying those genes active in commensal microbiome and host activities.

## Materials and methods

### Ethics statement and subject recruitment

The experimental human protocol was approved by the University of Illinois and Texas A&M University Institutional Review Boards and informed consent was obtained from parents prior to participation in the study. Details of the study admission criteria and protocols have been previously described [12]. Healthy, full-term infants who were exclusively breast-fed or fed commercially available infant formula (Enfamil LIPIL, Mead Johnson Nutrition, Evansville, IN, USA) and medically certified as healthy were eligible for enrollment into the study. For each infant in the study, stool samples were collected at three months after birth.

### Isolation of stool DNA

Genomic DNA was extracted using a modification of the method of Yu and Morrison [40]. Deviation from the protocol included the use of glass beater steps. Briefly, 250 mg (wet weight) of feces was weighed into a 2.0 ml tube containing glass matrix E (MP Biomedicals, Solon, OH, USA). Lysis buffer (1 ml; 500 mM NaCl, 50 mM Tris-HCl, 50 mM EDTA, 4% sodium dodecyl sulfate) was added to the tube and shaken for 30 s. Samples were then incubated at 70°C for 15 minutes. After centrifugation at 16,000 *g *for 5 minutes, supernatants were collected into 2.0 ml tubes. Lysis buffer (300 µl) was subsequently added and the above steps were repeated. Nucleic acids in the supernatant were precipitated sequentially with ammonium acetate and isopropanol, and dissolved in TE buffer. The precipitated nucleic acids were then treated with DNase-free RNase, proteinase K, and further purified on a QIAamp spin column from a QIAgen DNA Mini Stool Kit (Qiagen, Valencia, CA, USA). DNA quality was checked on 1% agarose gels followed by ethidium bromide staining. DNA from three to four extractions per sample was pooled and its concentration quantified using a NanoDrop 1000 spectrophotometer (NanoDrop Technologies, Wilmington DE, USA).

### Sequencing of gut microbiomes

DNA from fecal samples was submitted to the high throughput sequencing and genotyping unit at the Keck Center for Comparative and Functional Genomics, University of Illinois. Samples were sequenced using a 454 Life Sciences Genome Sequencer FLX with GS FLX Titanium series reagents (Roche, Nutly, NJ, USA). Briefly, DNA was fractionated (fragments of 500 to 800 bp) and polished. Subsequently, barcodes containing adaptors A and B were ligated to the ends according to Roche's instructions. Adaptor B contained a 5'-biotin tag to immobilize the DNA library on streptavidin beads. After nick repair, the non-biotinylated strand was released and used as a single-stranded template DNA (sstDNA) library. Library quantity was assessed using Qubit reagents (Invitrogen, CA, USA) and pooled to equal molarity. The optimal level of DNA for emulsion PCR was determined by titration. Beads were loaded onto a PicoTiterPlate device for shotgun sequencing. Signal processing was performed using Roche software.

### Host gut mRNA transcriptome analysis

From each subject, poly A^+ ^RNA was isolated from feces as previously described [12,41]. Due to the high level of bacterial RNA in fecal samples, poly A^+ ^RNA was isolated in order to obtain a highly enriched mammalian RNA population [12]. In addition, an Agilent 2100 Bioanalyzer was used to assess integrity of exfoliated cell poly A^+ ^RNA and quantification was performed on a NanoDrop Spectrophotometer. Samples were processed in strict accordance to the CodeLink™ Gene Expression Assay manual (Applied Microarray, Tempe, AZ, USA) and analyzed using the Human Whole Genome Expression Bioarray as we have previously described [13].

The microarray data have been previously processed and analyzed [12]. Technical errors in the probes were relatively rare, with approximately 2.5% of the probes being flagged. Nonetheless, thorough quality control processing resulted in 16,767 probes available for analysis. The log base 2 transformed expression data were normalized using two methods, standard loess normalization [42] as shown in Additional file 2, and a weighted median adjustment method [12]. Subsequently, and based on our findings in the microbiome sequence data, two data sets were constructed using curated gene lists based on literature reviews, functional gene assignments from PANTHER biological processes [43] and DAVID [44,45]. Using this prior knowledge, discrete sets of biomarkers (genes) known to be involved in intestinal biology (459) and immunity and defense (660) (see Additional files 8 and 9) [12,13] were generated. Focusing and targeting the scope of the data under consideration in a biologically meaningful way (i) reduces the dimension of the data being analyzed and protects against extensive multiple testing, (ii) allows for exhaustive examination of all small feature subsets (all three-gene sets) and thereby avoids feature selection, which is known to be highly unreliable in small sample settings [46], and (iii) allows for computational tractability and analysis feasibility. GO enrichment analyses were performed using the GO Fat gene ontology functional annotation tool [16], available on DAVID [44,45]. The expression values for enriched gene subsets were assessed using a permutation test and corrected for multiple testing discovery rate correction (FDR) [15].

### Metagenomic data analysis

Shotgun 454 read data were preprocessed in the following manner: (i) low quality reads were removed if the read mean Phred value was <20 and/or when two or more consecutive nucleotides exhibited a Phred value <20; (ii) reads were clustered using CD-HIT-454 [47] at 100% removing duplicates; (iii) the remaining shotgun sequences were analyzed using the MG-RASTv2 pipeline and the phylogenetic distribution and metabolic functional composition of the samples were profiled [48]. Representative MG-RAST sample statistics are shown in Additional file 5. Taxonomic classifications were assessed in two ways. First, identifiable 16S fragments in the shotgun sequences were used to align to the GreenGenes small subunit rRNA database [49]. Second, PhymmBL [17] was used as an additional metagenomic phylogenetic classification tool. PhymmBL uses BLAST and interpolated Markov models to taxonomically classify DNA sequences, including reads as short as 100 bp. In addition, to filter out possible human contamination from the reads, BLAST was used to compare all reads to the human genome (Genome Reference Consortium assembly, version 37, 2009 [50]). Any full length reads that were 100% identical to the human reference genome were discarded. Reads that were 100% identical, but whose length was under 80 amino acids or did not share a full-length alignment with the human reference sequence were not discarded. Between 0 and 13,222 reads were discarded from each sample. The percentage of discarded reads did not exceed 4.3% (13,222 discarded from sample 6) and in all other samples was lower than 0.4%.

Microbiota functional characteristics in BF and FF infants were compared. Additional file 10 provides a breakdown of the average number of reads across samples that were mapped to functional SEED categories. Because of the hierarchical structure of the SEED classification system, aggregating reads into coarser classifications provided for a more informed analysis. For SEED level 2 classifications, approximately 25% of the functional classifications exhibited an average number of 200 reads across samples. At SEED level 3, approximately 6% of the functional classifications had an average number of 200 reads. Subsequently, comparisons between functional categories were carried out subject to the following restrictions: SEED level 1 functional categories were compared if all the FF or BF samples had at least 200 reads from each sample and at least 2% of the overall number of reads of each sample; SEED level 2 functional categories were compared if all the FF or BF samples had at least 100 reads from each sample and at least 0.5% of the overall number of reads of each sample; SEED level 3 functional categories were compared if all the FF or BF samples had at least 50 reads from each sample and at least 0.1% of the overall number of reads of each sample.

### Gut metagenome and host transcriptome data integration

In order to take into account multivariate structure when assessing and ranking genes, we analytically quantified the multivariate relationships between the metagenomic and transcriptomic data. CCA was used to uncover the multivariate structure between the metagenome and host transcriptome data sets, which is discussed in more detail in Additional file 11[51]. CCA is a multivariate analysis method and provides measures of the strength - that is, canonical correlations - of multivariate association between variable sets as well as a means to interpret the role of the variables in terms of the underlying multivariate relationship [21-23]. The implicitly linear relationship embedded in CCA is targeted at the simplest first-order relationships that might be detectable between two data sets. Certainly, non-linear relationships are possible, and will not be detected by CCA methodology. However, such relationships are extremely difficult to estimate without large sample sizes, which are difficult to obtain in clinical settings involving infants, and thus we did not attempt to capture them given the small sample size of our current cohort. Since the CCA method is based on an estimate of the covariance matrix between the two variable sets, it is unreliable when the number of variables is large relative to the number of samples being used to estimate the covariance structure. Because of the limited number of subjects (six per treatment group), it was not possible to exhaustively examine all the microbiome and transcriptome outcomes simultaneously. Therefore, we repeatedly applied CCA to all subsets (of size three) of host gene expression variables combined with the metagenomic data (three variables). We refer to the subsets (of size three) of the gene expression variables as gene triples. By analyzing all gene triples in turn with the virulence characteristics, we examined the multivariate structure between the gut metagenome and host transcriptome in a piecewise, sub-dimensional manner. CCA results using either the loess normalization method or the weighted median adjustment normalization method were very similar (data not shown). Thus, only loess normalized data are presented.

As a result of our preliminary analysis of the metagenomic data, we targeted the SEED level 2 virulence characteristics for integration with the presumed relevant host gut gene expression data (immunological and defense genes as well as intestinal biology genes as described in the 'Host gut mRNA transcriptome analysis' section above). Four ('resistance to antibiotics and toxic compounds', 'Type III, Type IV, ESAT secretion systems', 'iron scavenging mechanisms', and 'virulence') out of thirteen ('invasion and intracellular resistance', 'prophage, transposon', 'toxins and superantigens', 'Type VI secretion systems', 'detection', 'pathogenicity islands', 'regulation of virulence', 'adhesion', and 'posttranslational modification' in addition to the preceding four categories) SEED level 2 virulence categories had more than ten sequence reads for each sample. We subsequently discarded the catch all 'virulence' category and used 'resistance to antibiotics and toxic compounds', 'Type III, Type IV, ESAT secretion systems', and 'iron scavenging mechanisms' as our so called 'virulence characteristics'. Each sample had more than 30 sequence reads representing each category and more than 50 sequence reads for all samples in either BF or FF groups. The average number of sequence reads was 245 over all categories and infants. Read count proportions were ultimately used in the CCA analysis. For integration with the virulence variables, we used the 100 of 660 immunological and defense genes and the 100 of 459 intestinal biology genes that had the smallest *P*-values. This was done to avoid a computationally prohibitive combinatorial explosion in the number of gene triples to be analyzed. The overall result of our approach was a list of 'best' host genes (out of those considered), that is, those showing the strongest empirical evidence of a relationship with the gut metagenome as judged by multivariate association and structure.

With regard to mathematical modeling, there is some similarity between CCA and principal components analysis (PCA). PCA is frequently used in high dimensional settings to uncover structure in the data, perhaps in conjunction with clustering methodologies, and to generally reduce data dimensionality. While there are slight differences in the mathematical optimization specification of PCA and CCA, they perform highly related analyses. The primary advantage of CCA in the present setting, however, is that it is specifically designed to uncover the multivariate structure between two distinct data sets. PCA makes no such prior distinction between data sets and thus does not specifically target the multivariate structure between two distinct data sets. We initially explored the use of PCA, but found CCA more adequately suited to the primary task of data integration. Additional file 12 shows the amount of variation explained by the first and second principal components for each gene triple/metagenome set examined. Sets characterized by only a few principal components would be expected to be potential candidates for strong performance under CCA; however, since the principal components in PCA do not necessarily target the underlying relationship between gene triples and the metagenome, they may instead identify factors influencing only gene triples or only the metagenome.

### Data deposition

The raw metagenome sequence data minus human-identical sequences are available at the European Bioinformatics Institute's Short Read Archive (study accession number: ERP001038). The human microarray data discussed in this publication have been deposited in NCBI's Gene Expression Omnibus [52] and are accessible through GEO Series accession number GSE31075.

## Abbreviations

BF: breast-fed; CCA: canonical correlation analysis; FDR: false discovery rate; FF: formula-fed; GO: Gene Ontology; LDA: linear discriminant analysis; PCA: principal components analysis.

## Competing interests

The authors declare that they have no competing interests.

## Authors' contributions

SS performed the data analysis and wrote the paper. IF assisted in data analysis design, performed the data analysis and wrote the paper. IVI assisted with experimental design and analyzed the data. LAD performed genomics assays. JSG performed genomics assays. DBD designed the study. DH performed data analysis. MW performed genomic assays. SMD designed the study and wrote the paper. RSC designed the study, assisted with data analyses and wrote the paper. All authors have read and approved the manuscript.

## Supplementary Material

Additional file 1 **Figure S1**. Overview of the analysis pipeline. **(a) **Stool samples were obtained from six breast-fed and six formula-fed infants. **(b) **Gut microbial DNA and host gut-epithelial mRNA were isolated and sequenced/hybridized. **(c) **Microbial DNA sequence was analyzed for functional content and taxa using MG-RAST and PhymmBL; gut epithelial mRNA was analyzed for eukaryotic gene function using microarray. **(d) **Significant multivariate correlations between gut-epithelium mRNA expression and metagenomic DNA frequency were determined using multivariate canonical correlation analysis (CCA) repeated on subsets of host gene expression data. Click here for file

Additional file 2 **Figure S2**. Original log 2 transformed raw CodeLink microarray data shown in an MA-plot. Upper panel: the x-axis shows the average of the average gene expression of BF and FF infants for each probe. The y-axis shows the difference between the two averages. The color bar shows the count density of the plotted data. BF samples exhibited a systematically higher gene expression level relative to FF samples. Lower panel: loess normalization of the original log 2 transformed raw CodeLink microarray data. This normalization procedure corrected for the systematic increase in BF gene expression relative to FF gene expression seen in the upper panel. The data were adjusted by the loess fit (blue line) shown in the upper panel. Click here for file

Additional file 3 **Table S1**. Host GO enrichment analysis. Click here for file

Additional file 4 **Figure S3**. Phyla distribution using 16S rRNA analysis (top) and PhymmBL classification of all reads (bottom). X-axis: sample numbers 1 to 6 BF, 7 to 12 FF. Y-axis: percentage of total assigned reads. See Additional file 8 for number of assigned reads. Click here for file

Additional file 5 **Table S2**. Counts of mapped microbiome sequences. Click here for file

Additional file 6 **Figure S4**. Example of canonical correlations of random gene sets. Analogous to the random gene set shown in Figure 4. Random (1,000) gene sets were sampled and analyzed. The first 5 of 1,000 are shown. Click here for file

Additional file 7 **Figure S5**. Example of the best performing genes in random gene sets. These data are analogous to the random gene set shown in Figure 5. Random (1,000) gene sets were sampled and analyzed. The first 5 of 1,000 are shown. Click here for file

Additional file 8 **Data set 1**. Discrete sets of biomarkers (genes) known to be involved in intestinal biology (459). Click here for file

Additional file 9 **Data set 2**. Discrete sets of biomarkers (genes) known to be involved in immunity and defense (660). Click here for file

Additional file 10 **Table S3**. Breakdown of sequencing depth in terms of average number of reads across samples mapped to SEED categories. Click here for file

Additional file 11 **Supplemental protocol**. Canonical correlation calculations. Click here for file

Additional file 12 **Figure S6**. A principal components analysis (PCA) of the virulence characteristics combined with all host gene triples. Top panel: host intestinal biology genes. Middle panel: immunity and defense genes. Bottom panel: random genes. The plots show the proportion of variation explained by the first and second principal components versus the variation explained by just the second principal component. The analyses provide a characterization of a lower dimensional structure underlying the data. When combined with the virulence characteristics, the immunity and defense genes (middle panel) generally exhibit a simpler latent structure compared to the other gene sets (top and bottom panels), as judged by the slight northeast shift in the point cloud. While the latent structure identified by PCA need not reflect a relationship between the virulence characteristics and the host genes, it may, in which case the immunity and defense genes are slightly more promising as a set with respect to future canonical correlation analysis (CCA) aimed at uncovering simple and strong relationships between the metagenomic and host transcriptome data. In this way, PCA may be used as a screening device to identify promising gene triples for CCA analysis. Click here for file
