# Supplementary figures and images for: A metagenomic study of diet-dependent interaction between gut microbiota and host in infants reveals differences in immune response
Source: Genome Biol. 2012 Apr 30;13(4):r32. doi: 10.1186/gb-2012-13-4-r32 (PMC3446306; doi:10.1186/gb-2012-13-4-r32)

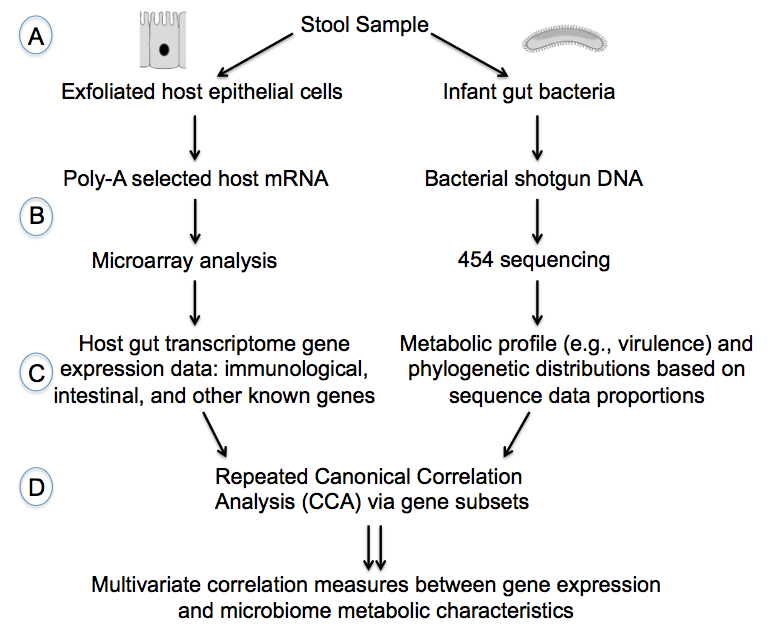

Supplement: Additional file 1 — Figure S1. Overview of the analysis pipeline. (a) Stool samples were obtained from six breast-fed and six formula-fed infants. (b) Gut microbial DNA and host gut-epithelial mRNA were isolated and sequenced/hybridized. (c) Microbial DNA sequence was analyzed for functional content and taxa using MG-RAST and PhymmBL; gut epithelial mRNA was analyzed for eukaryotic gene function using microarray. (d) Significant multivariate correlations between gut-epithelium mRNA expression and metagenomic DNA frequency were determined using multivariate canonical correlation analysis (CCA) repeated on subsets of host gene expression data. [file gb-2012-13-4-r32-S1.TIFF]

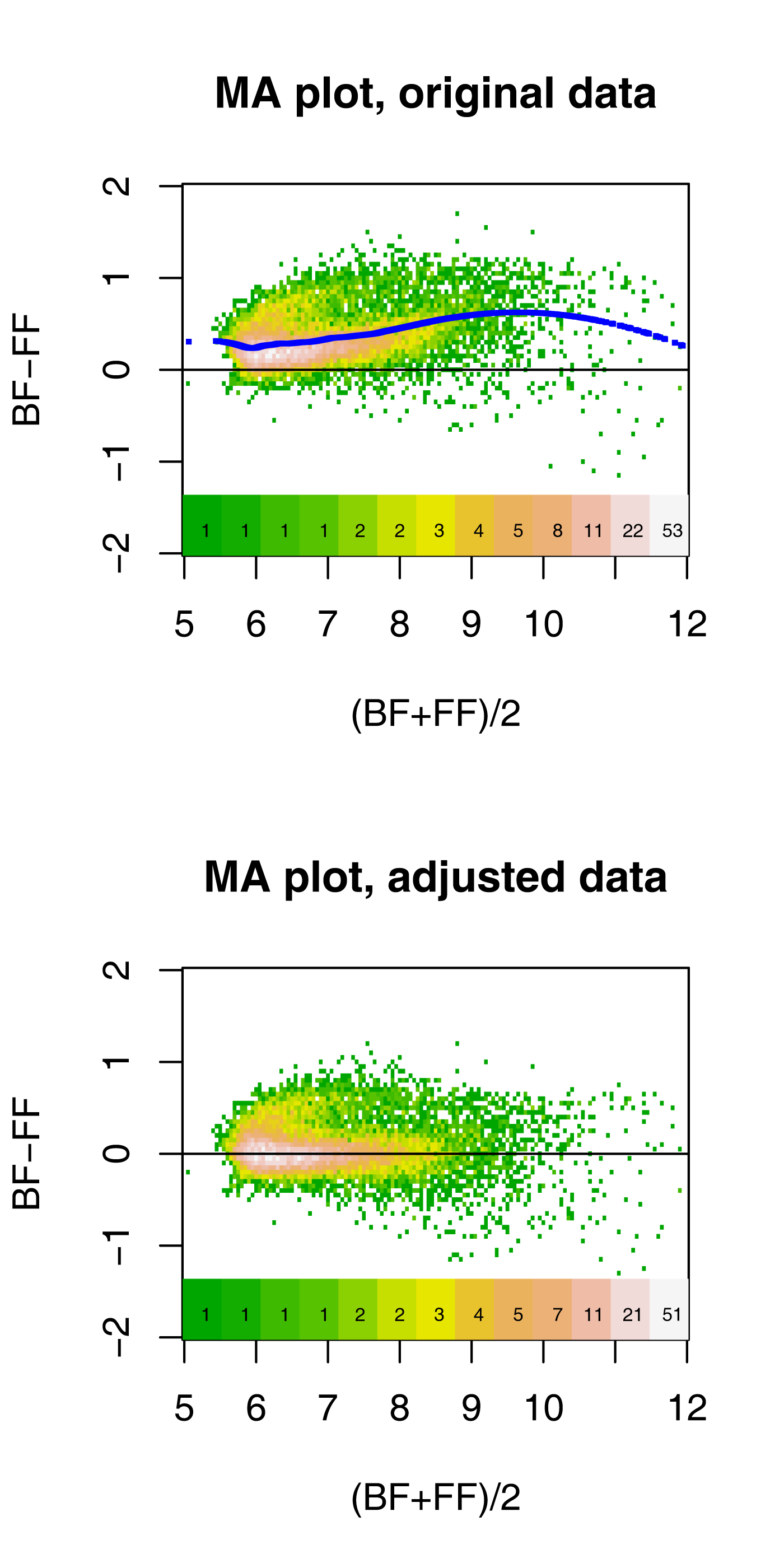

Supplement: Additional file 2 — Figure S2. Original log 2 transformed raw CodeLink microarray data shown in an MA-plot. Upper panel: the x-axis shows the average of the average gene expression of BF and FF infants for each probe. The y-axis shows the difference between the two averages. The color bar shows the count density of the plotted data. BF samples exhibited a systematically higher gene expression level relative to FF samples. Lower panel: loess normalization of the original log 2 transformed raw CodeLink microarray data. This normalization procedure corrected for the systematic increase in BF gene expression relative to FF gene expression seen in the upper panel. The data were adjusted by the loess fit (blue line) shown in the upper panel. [file gb-2012-13-4-r32-S2.TIFF]

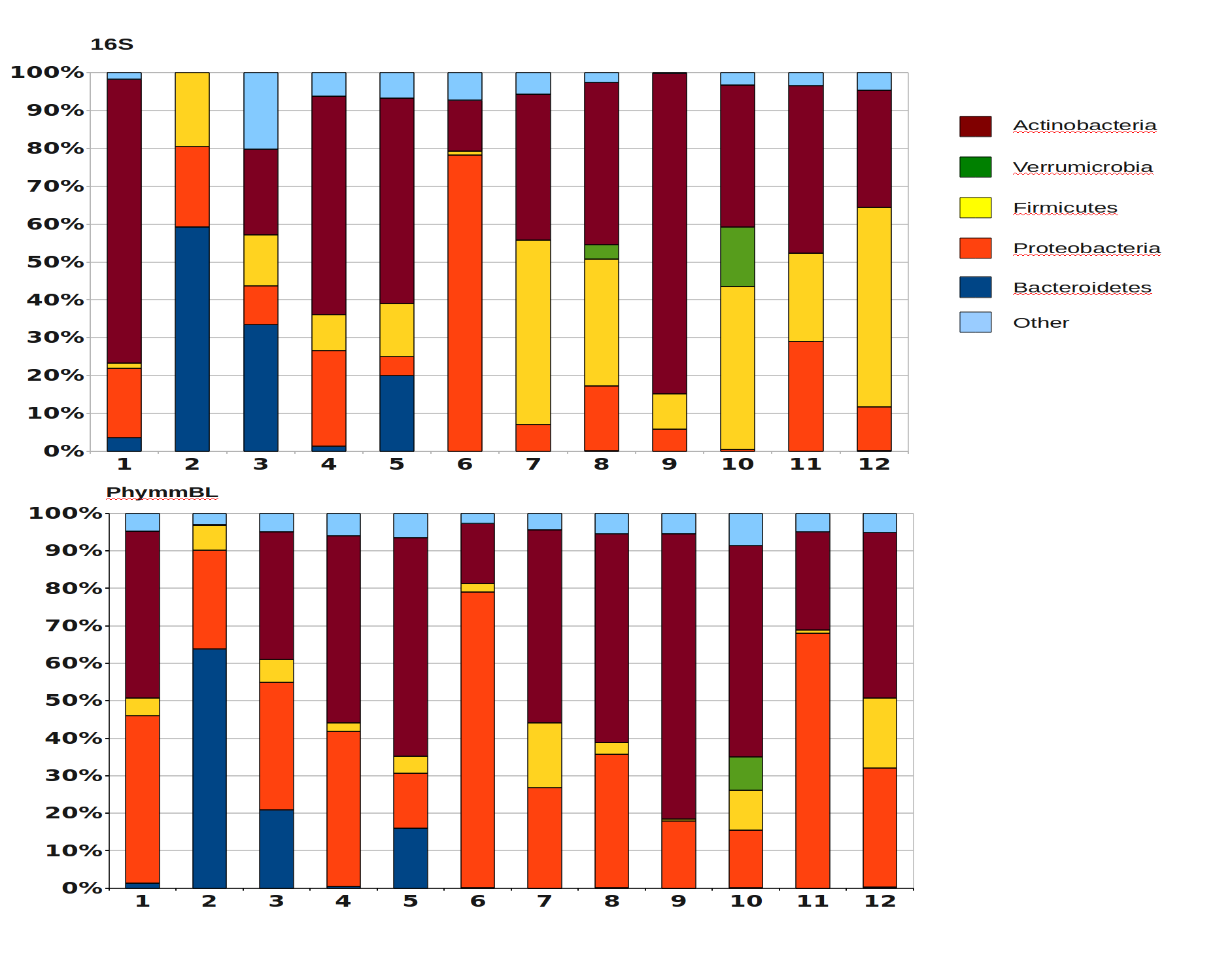

Supplement: Additional file 4 — Figure S3. Phyla distribution using 16S rRNA analysis (top) and PhymmBL classification of all reads (bottom). X-axis: sample numbers 1 to 6 BF, 7 to 12 FF. Y-axis: percentage of total assigned reads. See Additional file 8 for number of assigned reads. [file gb-2012-13-4-r32-S4.TIFF]

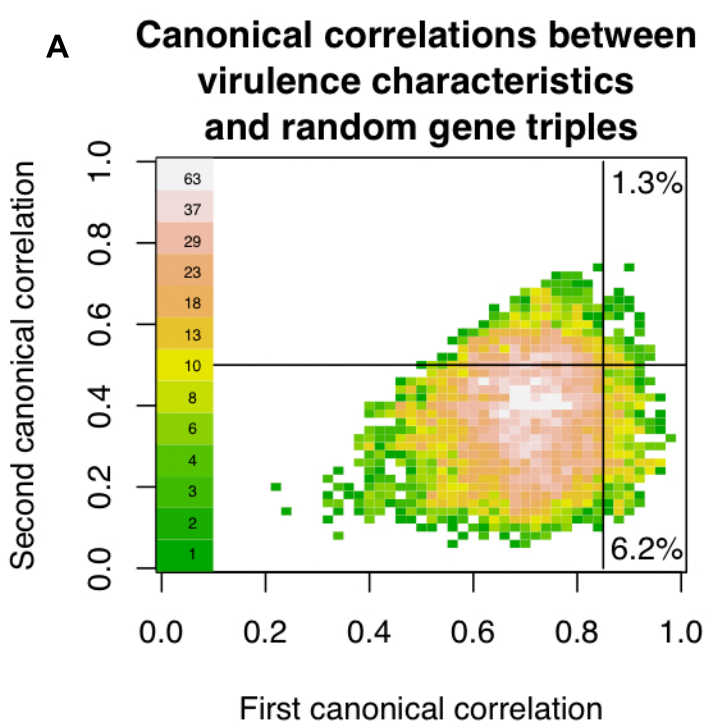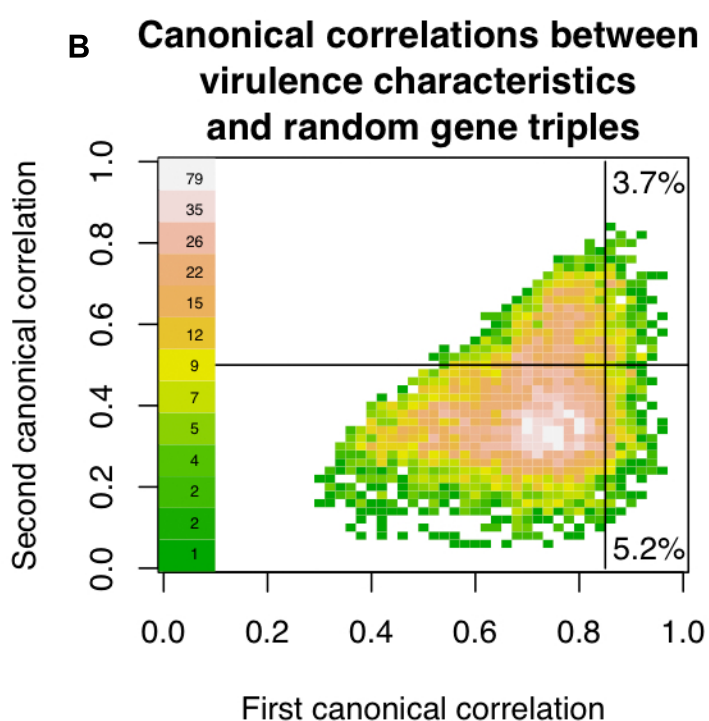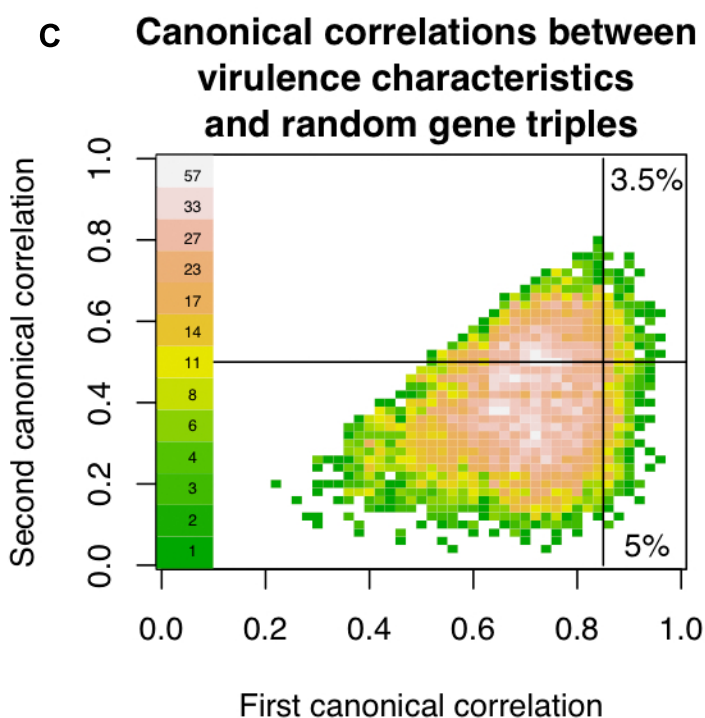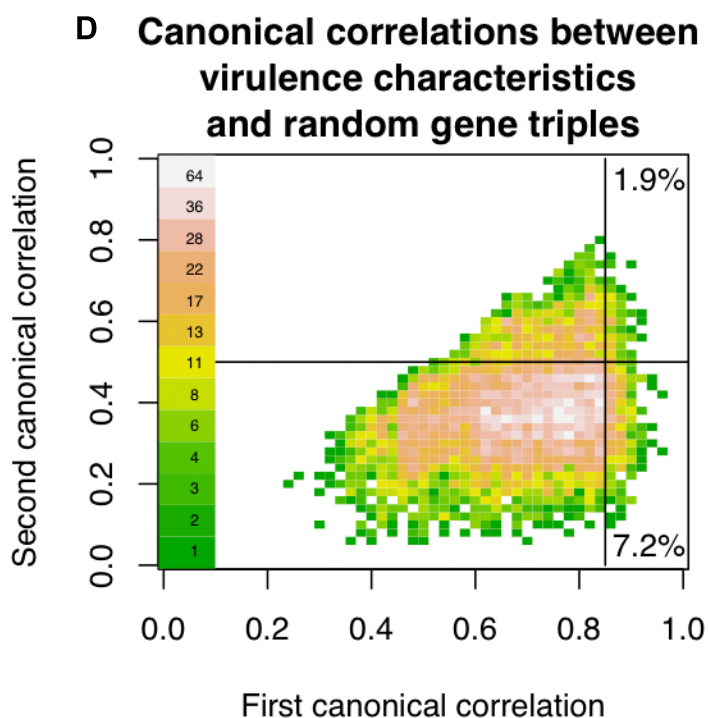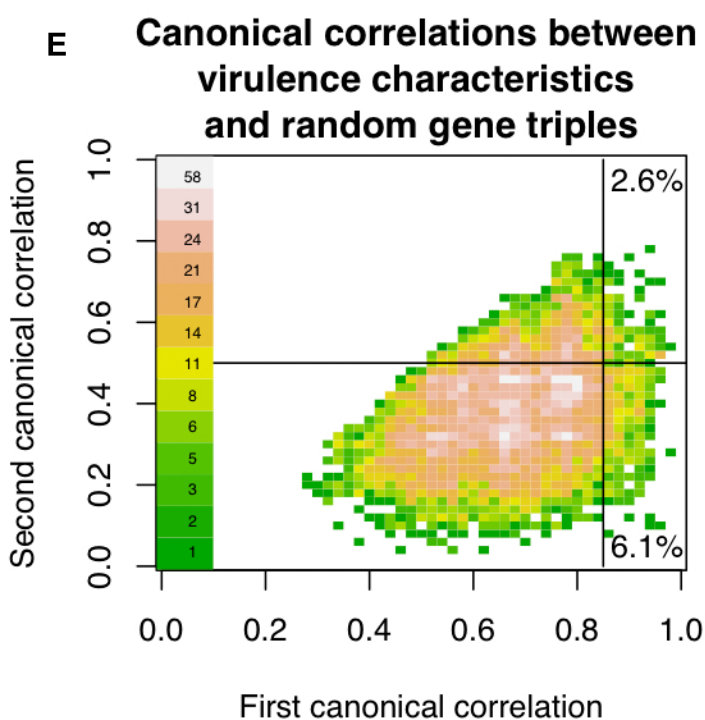

Supplement: Additional file 6 — Figure S4. Example of canonical correlations of random gene sets. Analogous to the random gene set shown in Figure 4. Random (1,000) gene sets were sampled and analyzed. The first 5 of 1,000 are shown. [file gb-2012-13-4-r32-S6.PDF]

A

Random genes

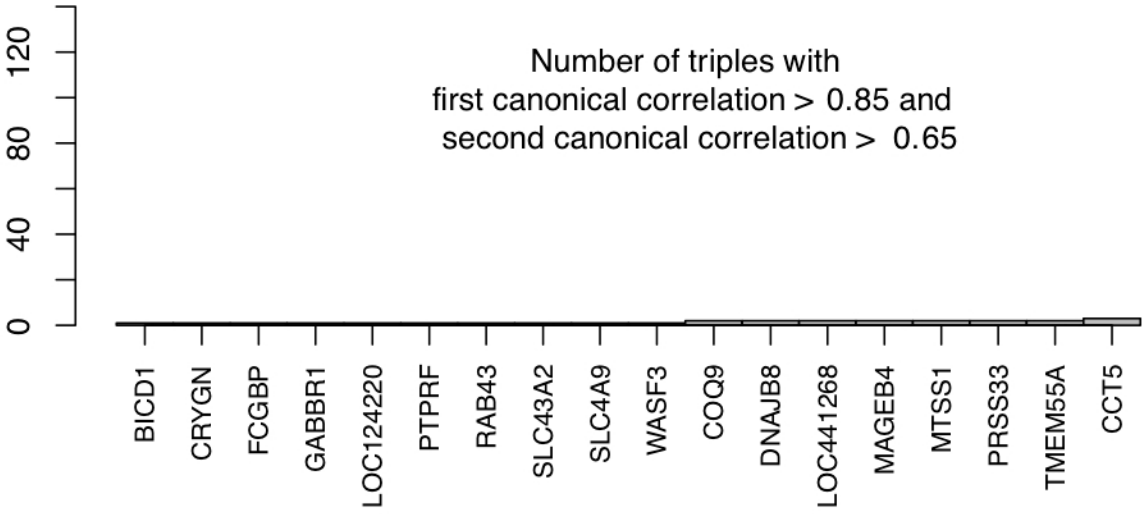

B

Random genes

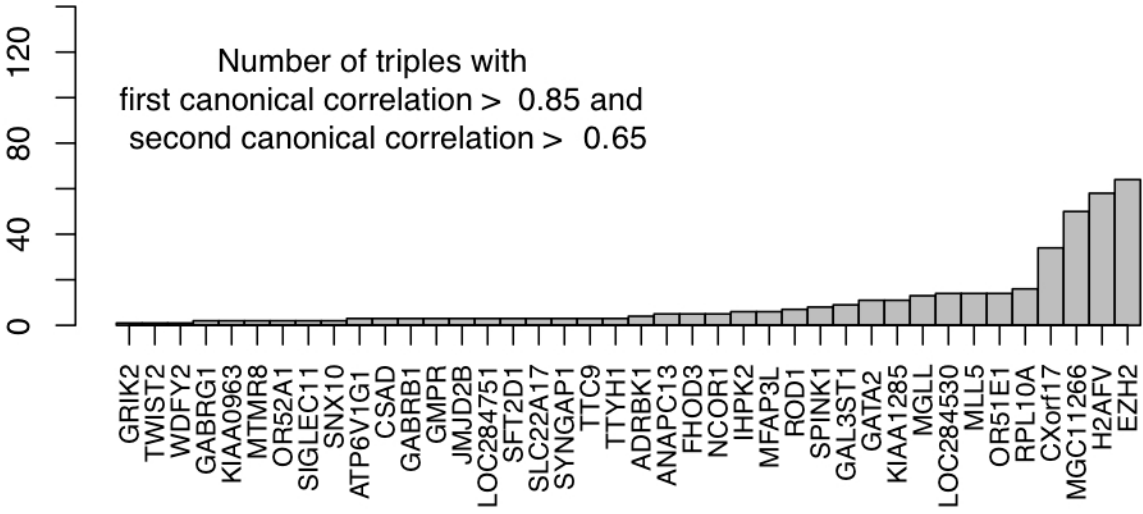

C

Random genes

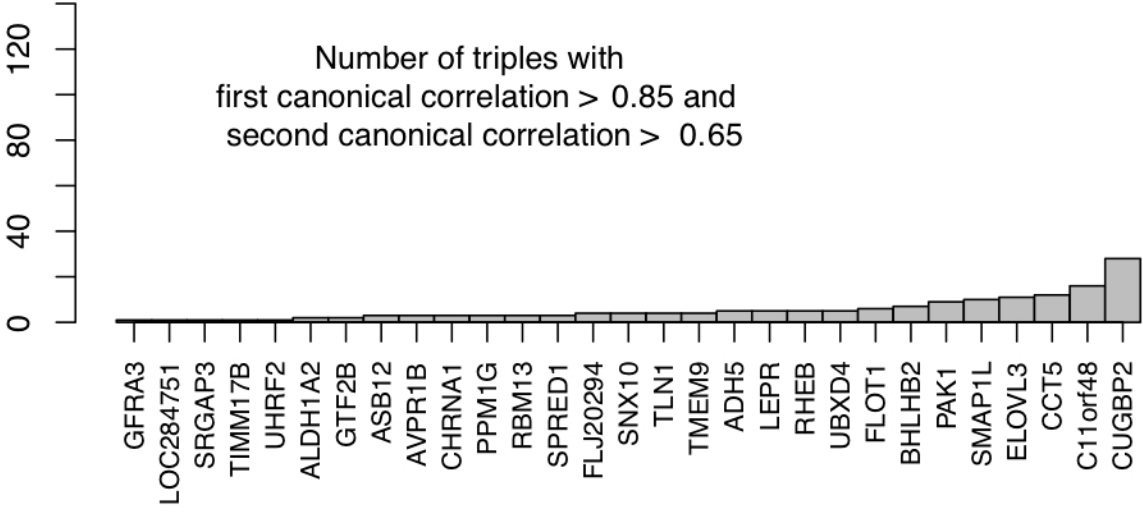

D

Random genes

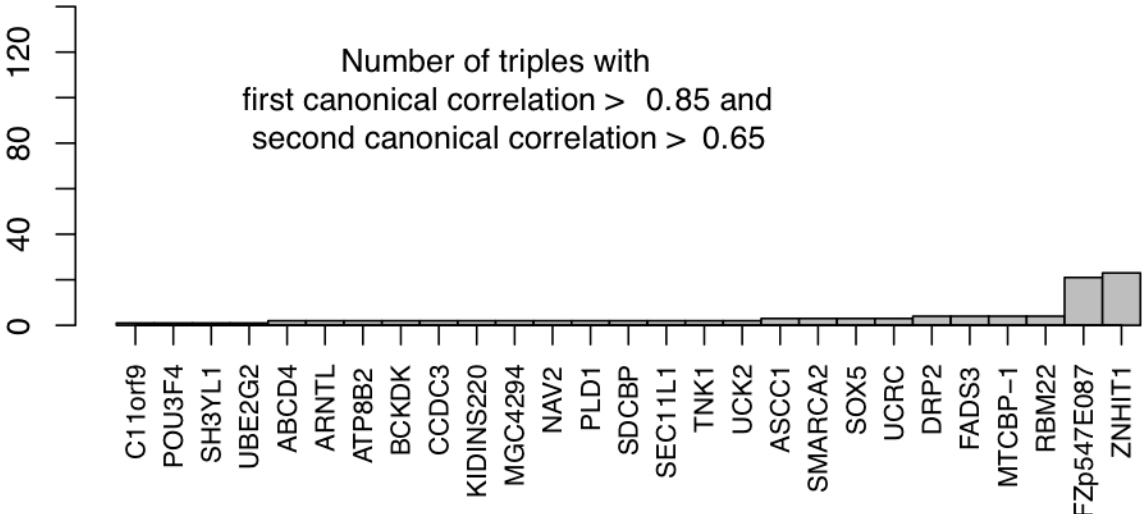

E

Random genes

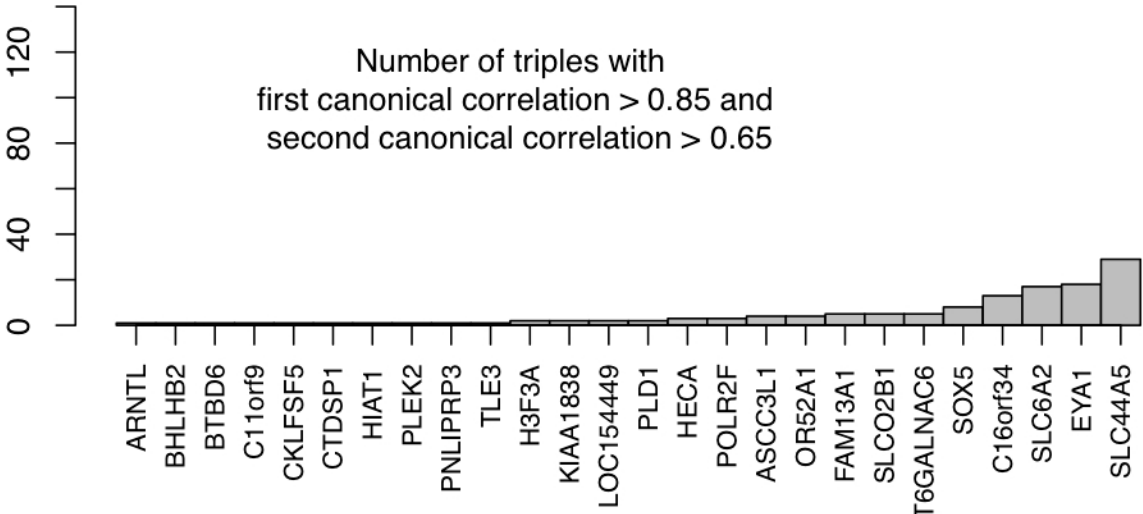

Supplement: Additional file 7 — Figure S5. Example of the best performing genes in random gene sets. These data are analogous to the random gene set shown in Figure 5. Random (1,000) gene sets were sampled and analyzed. The first 5 of 1,000 are shown. [file gb-2012-13-4-r32-S7.PDF]

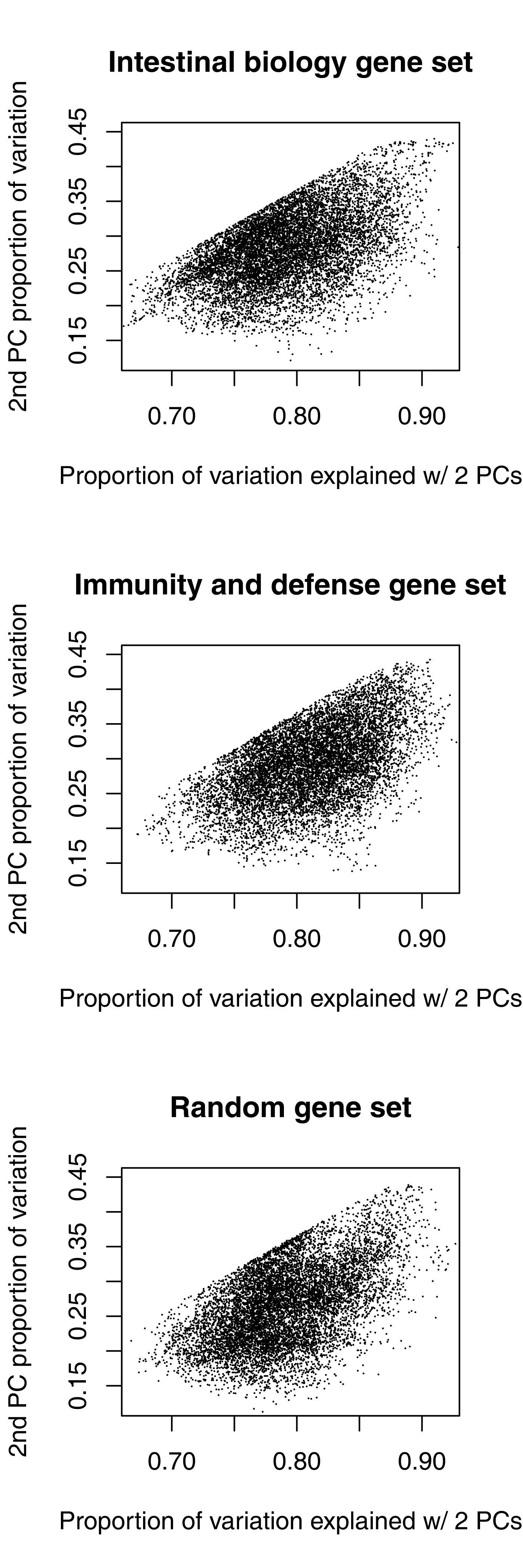

Supplement: Additional file 12 — Figure S6. A principal components analysis (PCA) of the virulence characteristics combined with all host gene triples. Top panel: host intestinal biology genes. Middle panel: immunity and defense genes. Bottom panel: random genes. The plots show the proportion of variation explained by the first and second principal components versus the variation explained by just the second principal component. The analyses provide a characterization of a lower dimensional structure underlying the data. When combined with the virulence characteristics, the immunity and defense genes (middle panel) generally exhibit a simpler latent structure compared to the other gene sets (top and bottom panels), as judged by the slight northeast shift in the point cloud. While the latent structure identified by PCA need not reflect a relationship between the virulence characteristics and the host genes, it may, in which case the immunity and defense genes are slightly more promising as a set with respect to future canonical correlation analysis (CCA) aimed at uncovering simple and strong relationships between the metagenomic and host transcriptome data. In this way, PCA may be used as a screening device to identify promising gene triples for CCA analysis. [file gb-2012-13-4-r32-S12.TIFF]
