# Supplementary material for: A metagenomic study of diet-dependent interaction between gut microbiota and host in infants reveals differences in immune response
Source: Genome Biol. 2012 Apr 30;13(4):r32. doi: 10.1186/gb-2012-13-4-r32 (PMC3446306; doi:10.1186/gb-2012-13-4-r32)
Supplement: Additional file 3 — Table S1. Host GO enrichment analysis. [file gb-2012-13-4-r32-S3.DOC]

**Table S1.** **Host gene ontology enrichment analysis.**

| **ID** | **Intestinal Biology Genes** | **Count** | **p-value** | **FDR** |
| --- | --- | --- | --- | --- |
| GO:0043066 | negative regulation of apoptosis | 24 | 3.04e-05 | 0.052 |
| GO:0060548 | negative regulation of cell death | 24 | 6.35e-05 | 0.109 |
| GO:0043069 | negative regulation of programmed cell death | 24 | 6.35e-05 | 0.109 |
| GO:0042981 | regulation of apoptosis | 33 | 8.62e-05 | 0.147 |
| GO:0010033 | response to organic substance | 29 | 0.000142 | 0.243 |
| GO:0043067 | regulation of programmed cell death | 33 | 0.000161 | 0.275 |
| GO:0010941 | regulation of cell death | 33 | 0.000161 | 0.275 |
| GO:0042127 | regulation of cell proliferation | 36 | 0.000744 | 1 |
| GO:0009611 | response to wounding | 26 | 0.00171 | 1 |
| GO:0007242 | intracellular signaling cascade | 27 | 0.00576 | 1 |
| GO:0006357 | regulation of transcription from RNA polymerase II promoter | 26 | 0.0139 | 1 |
| GO:0042592 | homeostatic process | 23 | 0.0163 | 1 |
| GO:0006350 | transcription | 33 | 0.023 | 1 |
| GO:0009891 | positive regulation of biosynthetic process | 25 | 0.0465 | 1 |
| GO:0010557 | positive regulation of macromolecule biosynthetic process | 24 | 0.0507 | 1 |
| GO:0031328 | positive regulation of cellular biosynthetic process | 24 | 0.0683 | 1 |
| GO:0010604 | positive regulation of macromolecule metabolic process | 26 | 0.0723 | 1 |
| GO:0051173 | positive regulation of nitrogen compound metabolic process | 22 | 0.0748 | 1 |
| **ID** | **Immunity and Defense Genes** | **Count** | **p-value** | **FDR** |
| GO:0008219 | cell death | 28 | 1.49e-06 | 0.00259 |
| GO:0016265 | death | 28 | 2.33e-06 | 0.00405 |
| GO:0007242 | intracellular signaling cascade | 37 | 2.87e-05 | 0.0498 |
| GO:0010941 | regulation of cell death | 36 | 3.19e-05 | 0.0554 |
| GO:0043067 | regulation of programmed cell death | 36 | 3.19e-05 | 0.0554 |
| GO:0042981 | regulation of apoptosis | 35 | 3.33e-05 | 0.0578 |
| GO:0009611 | response to wounding | 44 | 8.75e-05 | 0.152 |
| GO:0010033 | response to organic substance | 33 | 0.000106 | 0.185 |
| GO:0001775 | cell activation | 29 | 0.000116 | 0.202 |
| GO:0019220 | regulation of phosphate metabolic process | 27 | 0.000332 | 0.576 |
| GO:0051174 | regulation of phosphorus metabolic process | 27 | 0.000332 | 0.576 |
| GO:0042325 | regulation of phosphorylation | 26 | 0.00054 | 0.933 |
| GO:0007166 | cell surface receptor linked signal transduction | 53 | 0.000604 | 1 |
| GO:0006954 | inflammatory response | 28 | 0.00147 | 1 |
| GO:0006952 | defense response | 37 | 0.00201 | 1 |
| GO:0010604 | positive regulation of macromolecule metabolic process | 32 | 0.00325 | 1 |
| GO:0006350 | transcription | 26 | 0.0047 | 1 |
| GO:0042127 | regulation of cell proliferation | 30 | 0.00679 | 1 |
| GO:0009891 | positive regulation of biosynthetic process | 25 | 0.0329 | 1 |
| GO:0006955 | immune response | 40 | 0.08 | 1 |

Gene ontology (GO) enrichment was assessed using the DAVID bioinformatics (david.abcc.ncifcrc.gov) using the GO FAT vocabulary; 146 of the 459 *intestinal biology* genes and 191 of the *660 immunity and defense genes* exhibiting differential expression between BF and FF infants at the 0.2 FDR level. Enrichment was assessed relative to the background of the original genes, i.e., 459 *intestinal biology* genes and 660 *immunity and defense* genes (see **Additional files 8 and 9** for a complete listing). Therefore, the GO terms associated with *immunity and defense* do not appear here.
