# Supplementary material for: A metagenomic study of diet-dependent interaction between gut microbiota and host in infants reveals differences in immune response
Source: Genome Biol. 2012 Apr 30;13(4):r32. doi: 10.1186/gb-2012-13-4-r32 (PMC3446306; doi:10.1186/gb-2012-13-4-r32)
Supplement: Additional file 5 — Table S2. Counts of mapped microbiome sequences. [file gb-2012-13-4-r32-S5.DOC]

**Table S2.** **Counts of mapped microbiome sequences.**

| **ID** | **Diet** | **Reads** | **SEED** | **16S** | **PhymmBLL** | |
| --- | --- | --- | --- | --- | --- | --- |
| 1 | BF | 93,677 | 16,171 | 223 | 82,321 | |
| 2 | BF | 294,466 | 29,168 | 445 | 139,234 | |
| 3 | BF | 123,705 | 19,074 | 341 | 51,069 |  |
| 4 | BF | 199,013 | 37,308 | 596 | 141,649 | |
| 5 | BF | 207,765 | 38,471 | 776 | 143,075 | |
| 6 | BF | 239,566 | 41,559 | 382 | 123,291 | |
| 7 | FF | 123,124 | 19,101 | 462 | 77,937 | |
| 8 | FF | 146,350 | 31,040 | 691 | 109,531 | |
| 9 | FF | 253,407 | 17,289 | 649 | 134,590 | |
| 10 | FF | 196,856 | 38,848 | 258 | 106,814 | |
| 11 | FF | 258,644 | 28,551 | 763 | 141,280 | |
| 12 | FF | 151,811 | 34,365 | 460 | 122,592 | |

The Diet column indicates either formula (FF) or breast-fed (BF) stool samples. The Reads column lists the number of Roche 454 generated sequences. The SEED column represents the post-quality filter read counts: minimum acceptable sequence length, 100 not including barcodes; that were assigned SEED metabolic profiles with at least 80% identity. The microbial 16S rRNA gene column lists the number of 16S rRNA fragments of length at least 100 found in the shotgun sequencing reads with at least 96% identity. Taxonomic assignment to phyla using PhymmBL [11] was performed using a 0.9 phylum confidence score or better. PhymmBL uses BLAST and interpolated Markov models to taxonomically classify DNA sequences, including reads as short as 100 bp.
