# Supplementary material for: A metagenomic study of diet-dependent interaction between gut microbiota and host in infants reveals differences in immune response
Source: Genome Biol. 2012 Apr 30;13(4):r32. doi: 10.1186/gb-2012-13-4-r32 (PMC3446306; doi:10.1186/gb-2012-13-4-r32)
Supplement: Additional file 10 — Table S3. Breakdown of sequencing depth in terms of average number of reads across samples mapped to SEED categories. [file gb-2012-13-4-r32-S10.DOC]

**Table S3.** **Breakdown of sequencing depth in terms of average number of reads across samples mapped to SEED categories.**

| Average Number of Reads R | SEED 1 | SEED2 | SEED 3 |
| --- | --- | --- | --- |
| R < 10 | 7.4% | 21.0% | 38.0% |
| 10 ≤ R < 100 | 7.4% | 36.0% | 48.0% |
| 100 ≤ R < 200 | 7.4% | 18.0% | 8.9% |
| 200 ≤ R < 500 | 19.0% | 15.0% | 5.3% |
| 500 ≤ R < 1000 | 15.0% | 4.3% | 0.2% |
| 1000 ≤ R | 44% | 4.9% | 0.2% |
| Number of SEED Categories | 27 | 162 | 584 |

The proportion of SEED categories (for levels 1, 2, and 3) that achieved the specified depth cutoffs is shown. Since SEED is a hierarchical structure, aggregating to coarser SEED levels provided a more informed classification, although the interpretation of the categories becomes much more limited. As a result of the lack of sequencing depth available at the finer SEED classifications, analyses were confined to the higher SEED levels.
