# Supplementary material for: A metagenomic study of diet-dependent interaction between gut microbiota and host in infants reveals differences in immune response
Source: Genome Biol. 2012 Apr 30;13(4):r32. doi: 10.1186/gb-2012-13-4-r32 (PMC3446306; doi:10.1186/gb-2012-13-4-r32)
Supplement: Additional file 11 — Supplemental protocol. Canonical correlation calculations. [file gb-2012-13-4-r32-S11.PDF]

Supplemental CCA discussion

For two sets of standardized variables $(Y_{i1}^A, \cdots, Y_{ip_A}^A)$ and $(Y_{i1}^B, \cdots, Y_{ip_B}^B)$, e.g., gene expressions and metagenomic metabolic function profiles, with samples indexed by $i = 1, \cdots, n$, the sequential canonical correlations for $k = 1, \cdots, p = \min(p_A, p_B)$ are

$$\rho_k = \max_{a_k, b_k} \mathrm{cor}(A_{ik}, B_{ik})$$

subject to $\rho_k > \rho_j$ for $k < j$ and

$$\begin{aligned} \mathrm{var}(A_{ik}) &= 1 \\ \mathrm{var}(B_{ik}) &= 1 \\ \mathrm{cor}(A_{ik}, B_{ik'}) &= 0, \quad \forall k \neq k' \\ \mathrm{cor}(A_{ik}, A_{ik'}) &= 0, \quad \forall k \neq k' \\ \mathrm{cor}(B_{ik}, B_{ik'}) &= 0, \quad \forall k \neq k' \end{aligned}$$

where

$$\begin{aligned} A_{ik} &= \sum_{j=1}^{p_A} a_{jk} Y_{ij}^A \\ B_{ik} &= \sum_{j=1}^{p_B} b_{jk} Y_{ij}^B. \end{aligned} \tag{1}$$

A_{ik} and B_{ik} are called the kth canonical variates, and are created by the linear composites of Y_{ij}^A and Y_{ij}^B defined by the vectors $(a_{1k}, \cdots, a_{p_A k})$ and $(b_{1k}, \cdots, b_{p_A k})$, respectively. The sets of new variables $(A_{1k}, \cdots, A_{ip})$ and $(B_{i1}, \cdots, B_{ip})$ are, respectively, an affine transformation (projection) of $(Y_{i1}^A, \cdots, Y_{ip_A}^A)$ and $(Y_{i1}^B, \cdots, Y_{ip_B}^B)$ onto a new basis such that $p = \min\{p_B, p_A\}$ pairs of resulting variables are correlated and the remainder have zero correlation. That is, only (A_{ik}, B_{ik}), for $k = 1, \cdots, p$ have non zero correlation. In this way, the canonical correlations $(\rho_1, \cdots, \rho_p)$ show the optimal strength of (linear) relationship available between the two sets of variables $(Y_{i1}^A, \cdots, Y_{ip_A}^A)$ and $(Y_{i1}^B, \cdots, Y_{ip_B}^B)$. The linear composite parameters $(a_{1k}, \cdots, a_{p_A k})$ and $(b_{1k}, \cdots, b_{p_B k})$ provide a means to interpret the relationships in terms of the original variables in terms of the canonical correlations. CCA analysis is easily provided on the basis of the empirical covariance matrix of $(Y_{i1}^A, \cdots, Y_{ip_A}^A)$ and $(Y_{i1}^B, \cdots, Y_{ip_B}^B)$, and many statistical packages implement CCA as a prepackaged analysis capability.
